# Development and Evaluation of Chitosan-Based Food Coatings for Exotic Fruit Preservation

**DOI:** 10.3390/biotech12010020

**Published:** 2023-02-13

**Authors:** Juan Camilo Zárate-Moreno, Diana Marcela Escobar-Sierra, Rigoberto Ríos-Estepa

**Affiliations:** 1 Grupo de Bioprocesos, Departamento de Ingeniería Química, Facultad de Ingeniería, Universidad de Antioquia UdeA, Calle 70 No. 52–21, Medellín 050010, Colombia; 2 Grupo de Biomateriales, Programa de Bioingeniería, Facultad de Ingeniería, Universidad de Antioquia UdeA, Calle 70 No. 52–21, Medellín 050010, Colombia

**Keywords:** chitosan, fruit coating, fruit preservation, feijoa, fruit shelf-life

## Abstract

Chitosan has gained agro-industrial interest due to its potential applications in food preservation. In this work, chitosan applications for exotic fruit coating, using feijoa as a case of study, were evaluated. For this, we synthetized and characterized chitosan from shrimp shells and tested its performance. Chemical formulations for coating preparation using chitosan were proposed and tested. Mechanical properties, porosity, permeability, and fungal and bactericidal characteristics were used to verify the potential application of the film in the protection of fruits. The results indicated that synthetized chitosan has comparable properties to commercial chitosan (deacetylation degree > 82%), and, for the case of feijoa, the chitosan coating achieved significant reduction of microorganisms and fungal growth (0 UFC/mL for sample 3). Further, membrane permeability allowed oxygen exchange suitable for fruit freshness and natural physiological weight loss, thus delaying oxidative degradation and prolonging shelf-life. Chitosan’s characteristic of a permeable film proved to be a promising alternative for the protection and extension of the freshness of post-harvest exotic fruits.

## 1. Introduction

Feijoa (*Acca sellowiana* Berg) is a high value agricultural product that belongs to the family Myrtaceae and the subfamily Myrtroidea. It is a bright green-at-maturity fruit that is part of a special list of exotic fruits prompting increasing interest in international markets because of their nutritional and health benefits [1]. Feijoa crops have been developed and widely diversified in tropical climates; in Colombia, crops typically grow at altitudes from 2100 to 2700 m, with an average temperature of 13 °C [2]. Nonetheless, temperature, humidity, the presence of microorganisms, pathogens, and pests, interfere in the normal maturation process, thus affecting ripening and fruit flavor, and shortening shelf-life. The growing interest in the commercialization of feijoa supports the search for novel alternative approaches to its post-harvest handling and storage.

In general, the agricultural industry experiences limitations while marketing perishable products, e.g., fruits, mainly due to the presence of pollutants, microorganisms, and pests. Harvested agro-industrial products are largely susceptible to adverse storage and transport conditions. The post-harvest handling, transport and storage of fruit may be successfully improved by using biopolymer-based coatings, thanks to their antimicrobial and antifungal activities [3,4]. Other alternatives for storage involve cool storage conditions [5].

Chitosan is a natural polymer obtained from chitin deacetylation after successful demineralization, depigmentation and deproteinization processes. Sources of chitin are fungal species and the exoskeletons of insects and crustaceans. Chitosan is regarded as a non-toxic, biodegradable, and biocompatible polymer with a broad variety of applications [6]. Chitosan biofilms have been evaluated concerning their mechanical properties, permeability, surface assessment and antifungal and antimicrobial properties [7,8].

Crosslinking properties confers further chitosan capabilities and strength [9,10,11,12]. Chitosan biofilms have been successfully applied in conserving the natural characteristics of strawberries, apples, mangoes, and pears, which are continuously exposed to microorganisms and/or external contaminants [13,14,15].

In this study, chemical formulations for chitosan film preparations were proposed and tested for exotic fruit preservation, using feijoa as a case study. Furthermore, porosity and permeability, both fungal and bactericidal biofilm characteristics, were also examined.

## 2. Materials and Methods

### 2.1. Chitosan Extraction and Characterization

Chitin extraction from shrimp shells was carried out following standard protocols [16,17]. Initially, the starting material (shells) was washed to eliminate the adhered organic residues using potable water, and subsequently dried at 40 °C for 2 h. The cleaned material was further crushed and sifted until a suitable particle size (0.8–1.5 mm) was acquired. Following, the material was demineralized using hydrochloric acid (HCl) at 4%, treated at room temperature with a 1.5 solid: liquid ratio, under constant agitation for 2 h. Finally, deacetylation was carried out by hydrolysis of the acetamide groups in an alkaline medium at high temperature, and under constant agitation. Several samples were drawn to verify reproducibility.

Chitin deacetylation for chitosan production compelled a complete experimental design to obtain at least a standard quality material that can be used for fruit coating. This design considered three process variables (reagent concentration, temperature, and time of chitin exposure for further deacetylation), in a central composite experimental design. Statistical analysis (ANOVA and response surface optimization) was performed using Statgraphics^®^.

The obtained chitosan and its deacetylation degree was analyzed by Fourier transform infrared (FTIR) using transmission mode in a Shimadzu IRTracer-100 using the method of attenuated total reflection ATR in the range of 4000 to 500 cm^−1^.

### 2.2. Preparation of Chitosan Films

For chitosan biofilm preparation, process conditions were previously set and were based on the literature [18]; process variables such as acid concentration, temperature and time were defined through a central composite experimental design. Statistical analysis (ANOVA and response surface optimization) was performed using Statgraphics^®^.

The extracted chitosan powder was dissolved into an acetic acid solution at a 1:10 ratio and stirred at 350 rpm for 3 h, thus reaching complete homogenization; glycerol, as a plasticizer, was added at 1% *v*/*v*), and the resulting solution was stabilized for 15 min. Finally, 20 mL of the mixture were served on a 60 × 15mm plate and were isolated in an aeration chamber at room temperature for 24 h, in order to remove bubbles formed during the process. The films, formed after solvent evaporation, were later placed in desiccators prior to be used.

### 2.3. Characterization of Chitosan Films

#### 2.3.1. Film Thickness

Thickness is critical in determining other film properties such as permeability and mechanical strength. For film thickness determination, five (5) to eight (8) points were randomly selected and measured, thus covering the entire active film surface; for this, an analog micrometer Mitutoyo Quantu Mike (Mitutoyo, Aurora, IL, USA) was used. Further, statistics (mean +/− standard deviations) were applied to the measurements to determine the average film thickness.

#### 2.3.2. Water Vapor Permeability

Permeability was evaluated following ASTM E96/ASTM 96-05 standards. The tests were carried out in a controlled environment chamber at 25 °C and an average humidity of 37%. Chitosan film samples with an area of 0.1225 cm^2^ were sealed to the mouth of a container (glass tube) containing water. Then, the assemblage was placed within a controlled atmosphere. It was periodically weighed to determine the rate of vapor lost through the film, from water to the controlled atmosphere, according to the Gontard and Guilbert’s protocol [19].

To evaluate film performance over time, a linear regression analysis was performed, and, in turn, the rate of mass transfer was calculated according to Equation (1)

(1) $$WVT=\frac{dm}{dt}*\;\frac{1}{A}\;\;$$

where *WVT* is the water vapor transmission coefficient, $\frac{dm}{dt}$ is the differential mass/time and *A* is the area of the exposed film.

Then, the permeability was calculated by Equation (2):

(2) $$Permeability:\;\;\frac{WVT}{S\left(R_{1}-R_{2}\right)}$$

where *S* is the water vapor saturation pressure at room temperature, *R*_1_ is the relative humidity in the chamber, and *R*_2_ is the internal relative humidity of the test tube.

Finally, the permeance value multiplied by the film thickness allowed us to find the film’s permeability to water vapor. The experiment was performed in triplicate.

#### 2.3.3. Tensile Strength

Tensile strength is the maximum tensile stress that the film can sustain. It was evaluated based on the ASTM D882-12 standard, which describes the testing methodology for films of a thickness less than 1 mm. For this, a Universal Testing Machine (DIGIMESS) with 500 N load distributions and a 5 mm/min tension strain rate until break was used. Additionally, the film thickness was evaluated using a digital micrometer (Mitutoyo Quantu Mike). All tests were performed in triplicate.

#### 2.3.4. Analysis of Porosity

Morphological characterization of the membrane structure was carried out using scanning electron microscopy (SEM) (JEOL-JSM 6490 LV microscope), (Jeol, Tokyo, Japan) at an acceleration voltage of 20 kV for determining pore geometry and size, and the homogeneity of the film surface. Samples were fixed in a graphite tape and previously bombarded with gold ions (1.5 Å thick) to improve resolution, using a Sputtering Denton Vacuum Desk IV coupled to the scanning microscope.

### 2.4. Feijoa Coating Using Chitosan Films

A complete surface fruit pretreatment is necessary to remove potential pathogens and impurities that may alter the feijoa coating process conditions. Fruit samples were selected in such a way that they present neither slashes nor differences in harvesting time, and have similar bright conditions and appearance; this way, film performance during post-harvest, transport and/or storage could be assessed accurately. The fruit was cleaned with a 500-ppm sodium hypochlorite solution by immersion for 20 s, and washed with distilled water to remove the residual solution; residual distilled water was further removed with sterilized soft cloths.

Biofilm formation on the fruit surface was achieved by using the immersion technique (into the prepared chitosan solution). Pretreated and selected fruits were immersed for 10 s; chitosan excess was removed by gravity. Then, samples were dried for 8 min in a controlled chamber. Graphical recording of results was conducted for 15 days.

### 2.5. Microbiological Evaluation

The antimicrobial and antifungal properties of chitosan membranes have been reported for medical applications [20] and fruit coating [21]. Chitosan films were evaluated on feijoa; for this, a microbiological analysis was performed. Besides the barrier protections provided by the film, fruits were previously pretreated for the purpose of preserving freshness, aroma, and nutritional content. Selected fruits were at the same stage of maturity. Simultaneously, fruits without biofilm treatment were used as control. Samples were treated by triplicate.

The microbiological risk assessment for the diverse organisms used different selective culture media. Table 1 shows the most common microorganisms able to attack the fruit, based on their sugar content and moisture conditions.

The plate count method was used for quantification of microorganisms in samples. In the event of dense population of microorganisms, the method suggests performing log dilutions (base 10), as required. Surfaces were microbiologically evaluated for sampling procedure. For sampling, sterile distilled water, bacteriological peptone (Merk), sodium chloride (Carlo ERBA) and sterile swabs were used.

Peptone water (water, sodium chloride and peptone at a ratio 1:8, 5:1000) was prepared and sterilized for swab wetting. Moistened and sterilized cotton swabs were applied on the material surface for recovering the biomass and later releasing them on selected media. Experiments were performed in triplicate.

## 3. Results and Discussion

### 3.1. Chitosan Characterization

**Figure 1:**
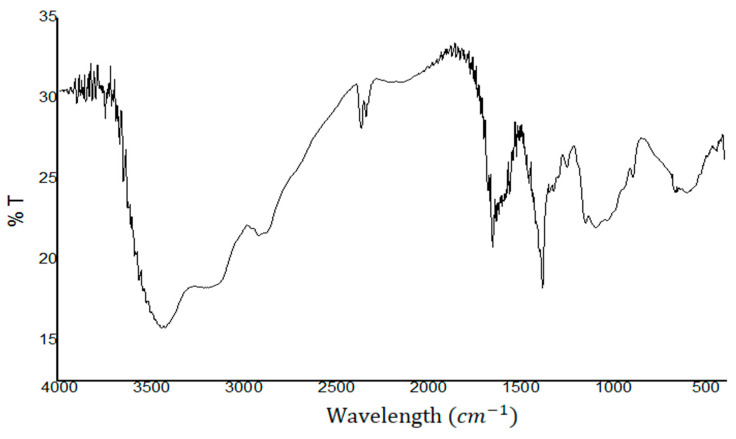
Fourier transform infrared spectrum of extracted chitosan.

Fourier transform infrared spectrum (FTIR) was used to identify the degree of deacetylation (DD) of the obtained chitosan samples 2 and 3. Figure 1 shows the corresponding FTIR spectrum. The spectrum reveals the presence of hydroxyl groups belonging to a broadband located at 3440 cm^−1^. Peaks of C-H stretching (3000–2850 cm^−1^), C=O stretching (amide I) (1650 cm^−1^), N-H bending (amide II) (1550 cm^−1^) and C-O (1280–1000 cm^−1^) were observed, as was also reported by other authors [22].

The deacetylation degree was calculated as described elsewhere [22]. The obtained percentages of deacetylation were 82.0%, 84.5% and 86.7% for the commercial sample, and for samples 2 and 3, respectively. These values are highly comparable to those required for chitosan industrial applications [23].

The chemical structure of chitosan lacks only the acetyl group, when compared to chitin. The hydrophobic nature of the acetyl group limits chitin solubility, while chitosan becomes soluble in aqueous solutions [24], considering the presence of an increased number of amino groups; at low pH, these amines are protonated and become positively charged. This condition makes chitosan a water-soluble cationic polyelectrolyte [25]. Further, the presence/absence of the acetyl group, because of the deacetylation process, may also affect the mechanical properties of chitin and chitosan.

The permeability of chitosan-based membranes is affected by the deacetylation degree; thus, low values of %DD are associated with low values of permeability and therefore a reduction in the percentage of oxygen that enters the fruit, which further may inhibit its ripening.

In contrast, the presence of acetyl groups contributes to the formation of stable H-bonds. Therefore, low tensile strength is expected for high vales of %DD [26,27]. Therefore, chitosan-based biofilm properties may prevent chitosan applicability in diverse fields.

### 3.2. Chitosan Biofilm Preparation

**Figure 2:**
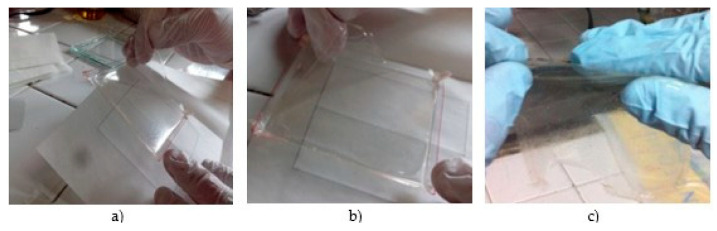
Chitosan films: (**a**) sample 2, (**b**) sample 3 and (**c**) commercial chitosan.

Figure 2 shows three elaborated films for comparison purposes. Figure 2a,b represent biofilms made with shrimp-extracted chitosan; Figure 2c shows a biofilm made with commercial chitosan.

A gradual improvement of film quality, regarding color, flexibility, and texture, was observed. From different experiments, it was noticed that a larger acetic acid content and/or higher temperatures did spawn coloring and poor mechanical properties in biofilms; therefore, the experimental setup was re-evaluated, thus overcoming film coloring and mechanical strength problems. For the case of edible films, there are special characteristics that need to be considered, among which are thickness, tensile strength, percent elongation, and transparency.

The most appropriate conditions for the elaboration of extracted chitosan films encompassed acetic acid (1% *v*/*v*), low chitosan concentration (1%) at a 1:10 solid: liquid ratio, and glycerol as a plasticizer (1.4%). The addition of the plasticizer was critical for improving film properties, mainly permeability and porosity; apparently, the plasticizer reduces intermolecular forces between the polymer chains, thus promoting an easy diffusion of water or oxygen for fruit respiration [28]. The advantages of using glycerol as a plasticizer in biopolymer synthesis are well known. Tarique J. et al. [29] did test its applicability to starch biopolymer development, observing that an increment in glycerol concentration caused an increment in film thickness and moisture content, though it also caused a significant reduction in tensile strength. In general, the controlled use of glycerol improves film properties.

### 3.3. Evaluation of Chitosan Films

Mechanical properties are important for edible films and coating since they contribute to a more versatile mechanical handling of fruits.

In Table 2, the average thickness for selected chitosan biofilm samples is shown. Thickness for tested biofilm formulations showed an acceptable variability of the diverse measurements; hence, samples were used for subsequent characterizations.

When compared with thickness of biofilms obtained from commercial chitosan, biofilms made of extracted chitosan were thinner on average. Yet, differences did not limit biofilm use and subsequent evaluation. The thickness of chitosan coatings is an important parameter considering the bright waxy green skin of feijoa fruits; as mentioned, the coating acts as a semipermeable barrier against oxygen, carbon dioxide and moisture; it also may reduce respiration and water loss, thereby counteracting the dehydration and shrinkage of feijoa fruits. The fruit can be consumed along with its skin, which is a little bitter but still contains plenty of nutraceutical qualities; therefore, the thinner the chitosan coating the better, considering the edible character of chitosan.

**Figure 3:**
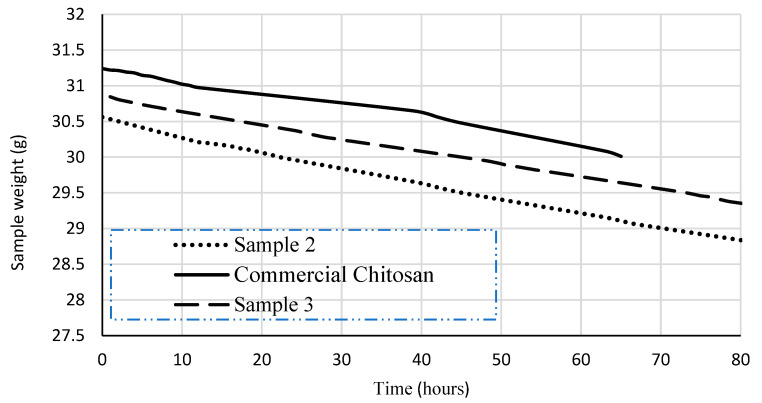
Weight loss for the assemblage using films made with commercial chitosan and extracted chitosan, in permeability experiments.

Regarding biofilm permeability, Figure 3 shows the permeability results for the commercial chitosan sample and the extracted chitosan samples. Assays were carried out on the films that showed the best results (qualitative), in terms of transparency, appearance and thickness. These characteristics would enable films to promote mass transfer in a controlled manner, thus reducing adverse effects on the fruits’ organoleptic properties.

As observed in Figure 3, the weight of the assemblage used for the permeability test, considering the different samples, showed a linear trend over time. Weight loss rate was slightly different among the tested samples. Permeability values for commercial chitosan samples ranged from 2.293 × 10^−9^ g/(m s Pa) and 3.382 × 10^−9^ g/(m s Pa).

These values were higher than those acquired for chitosan samples 2 and 3 (1.37 × 10^−9^ g/(m s Pa) and 1.53 × 10^−9^ g/(m s Pa), respectively). Still, permeability values for extracted chitosan samples were highly comparative to those reported for polymers used in food coating, e.g., (wheat) gluten (1.4–4.6 × 10^−9^ m.s.Pa) [30] and isolated soy protein (1.6–4.4 × 10^−9^ g/m.s.Pa) [31,32], and were higher than those informed for polyethylene (2.4 × 10^−13^ g/m.s.Pa) [33]. Hence, the obtained biofilms showed increased permeability, a property quite useful for respiration and oxygenation of coated fruits. Nonetheless, high permeability values may affect fruit oxygenation, thus hastening growth and physiological weight loss, and reducing the fruit’s shelf-life [28,34]. Weight loss might be the major determinant of the storage life and quality of fruits. The use of chitosan coatings significantly reduces the weight loss of fruits during storage, since it forms an additional barrier against water diffusion through the stomata [21]. These outcomes have been also observed in fruits such as papaya, strawberries, and cucumber and pepper [21].

**Figure 4:**
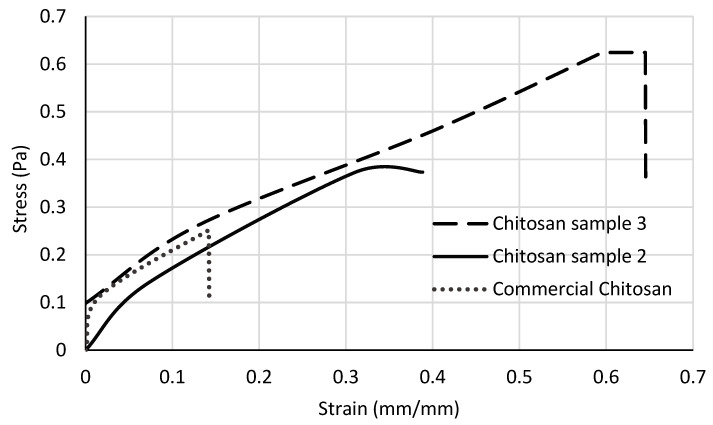
Stress–strain curve for chitosan biofilms.

Figure 4 shows the tensile test results for both the extracted and commercial chitosan films. These data were essential for the calculation of the biofilm strength to applied forces, the Young’s modulus, the biofilm tensile strength and the elastic limit of both extracted and commercial chitosan biofilms (Table 3).

As noted, sample 3 has the lower modulus of all samples tested (Table 3); this might be the result of its lower rigidity caused by the rendered deacetylation degree. It has been well documented that the higher the deacetylation degree, the greater the solubility and ease of crosslinking [28,35].

Clearly, the tensile strength of the extracted chitosan films was higher compared to that of the commercial chitosan sample (0.3733 Pa and 0.6244 Pa for samples 2 and 3, respectively, and 0.2514 Pa for commercial chitosan); it is expected that film’s tensile strength increases with increasing degree of deacetylation (%DD 82.1; 84.5 and 86.7 for commercial, sample 2 and sample 3, respectively) [35].

Moreover, it was observed that at higher %DD of chitosan, the resistance properties of the derived films seem to be favored; there was less rigidity and greater resistance to deformation. This behavior could be affected by parameters such as molecular weight, polymer–solvent interactions, and the presence of plasticizers [18].

**Figure 5:**
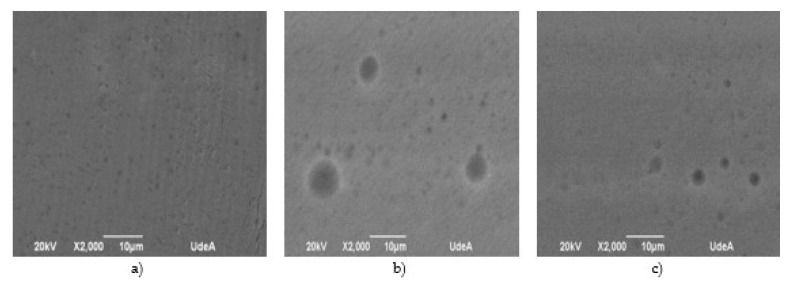
SEM micrographs for chitosan biofilms: (**a**) commercial, (**b**) sample 2 (**c**) and sample 3.

Figure 5 shows SEM micrographs for the evaluated samples: the chitosan commercial sample and the chitosan extracted samples.

Porosity is another important biofilm property; it is very critical for molecule transfer, molecular interactions, polymer relaxation and affinity. It allows water, or any other compound, to pass through. It is known that that low porosity values may not affect diffusion; molecular transport is limited only by polymer diffusion mechanisms. In this study, porosity for the obtained samples was smaller than 2 µm.

As pointed out, porosity changes with the degree of deacetylation for samples 2 and 3. Porosity values are the results of a combination of factors. The concentration of residual minerals in the sample might affect porosity of chitosan biofilms; pH values can also affect biofilm porosity, especially at the interval of 3 to 5 units. Under these conditions, solubility is affected, thus generating regions with weak bonds and altered porosity [36].

Though porous size differs between those on biofilms obtained with plasticizers and glycerol, permeability (5.17 × 10^−6^g/(m.h.Pa)) values are comparable to those reported by Wiles et al. [37] (7.41 × 10^−7^g/(m.h.Pa)), which suggest glycerol, as plasticizer, confers adequate properties for transport and controlled molecule release to the biofilm.

### 3.4. Feijoa Coating Using Chitosan Films

Chitosan biofilm coating for the protection of fruits may extend freshness and moisture content [8,38,39]. After a meticulous disinfection and a preliminary classification based on size, color, freshness and appearance, fruits were coated with chitosan biofilms, using the immersion technique. Skipping the initial disinfection step does leave fruits completely attacked by fungal and/or microbial agents. The presence of the coating has demonstrated an efficient control of microbial and fungal growth, hence preserving fruit shelf-life and freshness [25]. The chitosan biofilm properties contribute to an appropriate fruit ripening and homogeneous preservation as long as the coating process is successful. Coating can be applied either single or in a multilayer structure with stable performance [40].

**Figure 6:**
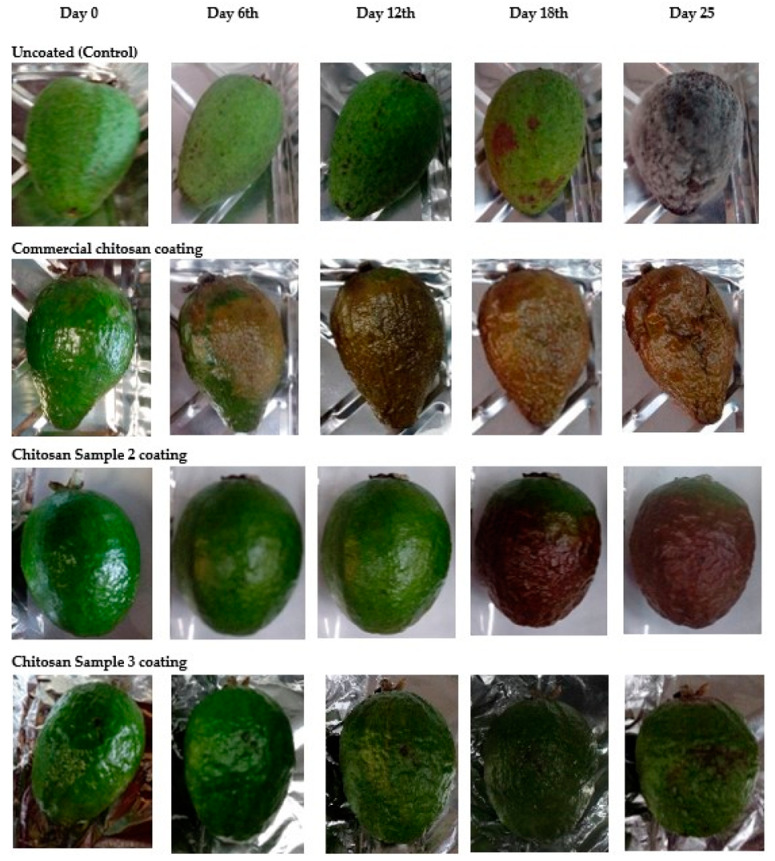
Fruit ripening with and without film coating for different coating samples.

In this study, experiments were run for fruits exposed to a previous disinfection process using commercial and extracted-chitosan films. Uncoated fruits were used as a control (Figure 6).

As observed in Figure 6, fruits without coating developed a massive fungal growth, whereas coated fruits were further preserved, considering the antifungal and antimicrobial chitosan properties. Chitosan-based coatings are known to be the best edible and biologically safe preservative for fruits and vegetables, since they have antimicrobial action and are biodegradable and nontoxic [41]. For chitosan-coated fruits, surfaces still preserved their luster and showed low variability and scarce grooves; the coating controlled water transpiration and loss of nutrients, thus enhancing resistance to external agents that may vary their maturation over time. Though it was not part of this study, it is expected that feijoa’s antioxidant properties were also preserved. In contrast, non-coated fruits showed an accelerated ripening process, besides to a manifest external-agent attack, mainly fungi and bacteria.

As pointed out, biofilm capacity for oxygen transfer depends on porosity, thickness, and permeability; also, it has been observed that the degree of chitosan deacetylation influences film permeability. Although the extracted chitosan films showed lower permeability than that of commercial chitosan films, oxygen transfers and fruit respiration were not compromised, hence rendering longer a shelf-life for coated fruits.

**Figure 7:**
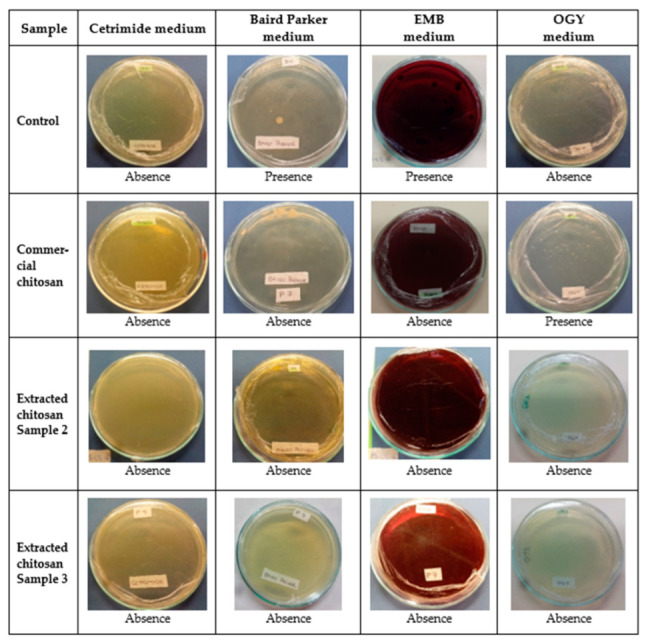
Microbial analysis of uncoated/coated feijoa after 15-day exposition.

Following the fruit coating experiments, microbiological samples were taken from disinfected coated fruit samples. Samples were taken at day zero and on the 15th day (Figure 7).

Feijoa samples (previously disinfected and provided with chitosan coatings) that exhibited microbial growth on a selective medium were microbiologically evaluated and quantitatively described by the plate-count method and Gram staining; these analyses provided data for colony forming units for each microorganism. Extracted chitosan biofilm sample 3 had no presence of fungi or microorganisms, as shown in Figure 7; this biofilm formulation might be recommended for the protection of feijoa fruits against microorganisms.

For biofilms of chitosan sample 2, colonies appeared on the Baird-Parker medium, which determines the presence of *Staphylococcus*; the presence of this microorganism was further validated by Gram staining.

Once microbial evaluation of Feijoa with and without chitosan coatings was performed, it was determined that the presence of microorganisms, on the first day, was reduced due to the fruit disinfection with sodium hypochlorite. On the 15th day, the presence of microorganisms was determined in three out of four samples evaluated; these were characterized by Gram staining and plate counts for each microorganism type, grown on selective media.

Colony-forming units in these selected media were larger for uncoated fruits, thus confirming the advantages of using biofilms for fruit protection [20,21]. Apparently, the polycationic nature of chitosan favors the antifungal and antimicrobial activities through a hitherto unclear mechanism of action between the microbial cell membranes and either chitosan amine groups or low molecular weight chitosan molecules that may penetrate [42].

Fruit samples with commercial chitosan-based coating were microbiologically evaluated; as observed (Figure 7), at 15th day, the OGY selective media was the only one that showed a colony forming units (fungus). For the remaining selective media, absence of colony units was observed after the 15th day of the experiment, thus confirming the potential of the antimicrobial and antifungal chitosan properties.

Regarding the quantification of microorganisms, samples were evaluated on plate count agar with different dilution factors, according to the concentration of colonies; the results are shown in Table 4 for commercial and extracted chitosan biofilms.

By day 15, colonies of microorganisms in samples without pretreatment increased; the presence of *E. coli* and *Staphylococcus* (>500,000 UFC/mL) in samples with commercial chitosan coatings was also observed. The extracted chitosan film 2 showed comparative UFV/mL values to commercial chitosan films for *Staphylococcus*. It was expected that the coatings would provide complete protection against Gram-positive and Gram-negative organisms, as well as fungi. Apparently, the antimicrobial properties of chitosan-based coatings were not that efficient, perhaps due to operational conditions. As an example, it has been reported that pH values interfere with chitosan adsorption rates and antimicrobial activity in *E. coli*; adsorption strongly increases with increasing pH [43]. Hence, finding the appropriate experimental conditions would greatly improve chitosan-based coating antimicrobial efficiency. Moreover, antimicrobial chitosan properties can be improved by blending synthetic and natural antimicrobial agents derived from essential oils with chitosan-based coatings. In this sense, some herb varieties, e.g., basil, oregano, and thyme, have been studied [44]. Chitosan treated with cotton fabric has also been shown to be effective in causing morphological changes and shrinking after contact with cell membranes of Gram-positive and Gram-negative bacteria. The forming layer blocks channels and prevents the transport of essential nutrients, causing severe damage and ultimately cell death [45].

Extracted chitosan biofilm 3 showed no UFC values. Seemingly, modifications on the cell surface did appear, thus altering the integrity of the cell membrane and interfering with nutrient transport and/or energy metabolism. This likely mechanism for effective antimicrobial activity has been hypothesized to involve electrostatic forces between the protonated amino groups and the negative residues at cell surfaces [43]. Further, researchers have also demonstrated that phosphoryl groups of the phospholipid component of the cell membrane are responsible for the electrostatic interaction with chitosan polycationic groups, which reinforce its antimicrobial properties [46].

## 4. Conclusions

In this work, the characteristics of chitosan as a permeable film were evaluated for the protection and extension of the freshness of post-harvested food. For the case of exotic fruits, chitosan biofilms achieved significant reduction and even zero growth of microorganisms and fungi in feijoa fruits coated with chitosan films. The obtained chitosan–membrane permeability allowed a proper oxygen exchange suitable for fruit freshness and normal physiological weight loss, thus delaying oxidative degradation and prolonging shelf-life.

However, it is known that chitosan films are permeable to water vapor, thus limiting their applicability, especially in moist environments. Researchers have dealt with this drawback by increasing film hydrophobicity using neutral lipids, fatty acid waxes and/or clay, the addition of cross-linking agents, and blending with proteins and/or polysaccharides. These approaches not only improve chitosan-based film permeability characteristics but also improve its mechanical properties. These strategies are the subjects of our current research on exotic fruit preservation. Based on our findings, we argue that chitosan-based protection is a worthy alternative for the preservation of valuable and exotic fruits.

## Figures and Tables

**Table 1:** Microbiological evaluation of specific organisms.

Cell Culture	Medium *	Gram Staining	Description
*Clostridium perfringes*	TSN	Gram-positive bacillus	Presence or absence
*Escherichia coli*	EMB	Gram-negative bacillus	Presence or absence
*Salmonella* sp.	XLD	Gram-negative bacillus	Presence or absence
*Pseudomona Aeruginosa*	Cetrimide	Gram-negative bacillus	Presence or absence
*Staphylococcus aureus*	Baird parker	Gram-positive coccus	Presence or absence
*Aspergillus niger—Candida albicans*	OGYE	Fungus	Fungus presence

* TSN: tryptone–sulfite–neomcyine Agar. EMB: eosin–methylene blue agar. XLD: xylose–lysine–desoxycholate agar. OGYE: oxytetracycline–glucose–yeast extract agar.

**Table 2:** Average thickness for chitosan biofilm evaluated.

Evaluation	Sample 2 Extracted Chitosan (cm)	Sample 3 Extracted Chitosan (cm)	Commercial Chitosan (cm)
1	0.00552	0.00652	0.02270
2	0.00522	0.00822	0.02202
3	0.00712	0.00582	0.02230
4	0.00542	0.00922	0.02103
Average thickness	0.00601	0.00852	0.02200
Deviation	0.001294	0.0019	0.00073
Confidence interval	0.000897	0.001326	0.0071

**Table 3:** Biofilms’ mechanical properties.

Sample	Elastic Limit (Pa)	Tensile Strength (Pa)	Young’s Modulus (Pa)
Commercial chitosan	0.1142	0.2514	1.1001
Extracted chitosan sample 2	0.1400	0.3733	0.9739
Extracted chitosan sample 3	0.2629	0.6244	0.7325

**Table 4:** Microbial counting at day 15 for different chitosan biofilm sources.

Feijoa Samples	EMB Cell Culture (UFC/mL)	Baird-Parker Cell Culture (UFC/mL)	Cetrimide Cell Culture (UFC/mL)	OGY Cell Culture (UFC/mL)
Uncoated	501,000	6,100,000	0	Several fungi
Commercial chitosan	0	561,000	0	0
Chitosan Sample 2	0	510,000	0	0
Chitosan Sample 3	0	0	0	0

## Data Availability

Not applicable.
